# Loci discovery, network-guided approach, and genomic prediction for drought tolerance index in a multi-parent advanced generation intercross (MAGIC) cowpea population

**DOI:** 10.1038/s41438-021-00462-w

**Published:** 2021-02-01

**Authors:** Waltram Ravelombola, Ainong Shi, Bao-Lam Huynh

**Affiliations:** 1 Department of Horticulture, University of Arkansas, Fayetteville, AR 72701 USA; 2 Texas A&M AgriLife Research& Extension, Vernon, TX 76384 USA; 3 Department of Nematology, University of California, Riverside, CA 92521 USA

**Keywords:** Plant breeding, Drought

## Abstract

Cowpea is a nutrient-dense legume that significantly contributes to the population’s diet in sub-Saharan Africa and other regions of the world. Improving cowpea cultivars to be more resilient to abiotic stress such as drought would be of great importance. The use of a multi-parent advanced generation intercross (MAGIC) population has been shown to be efficient in increasing the frequency of rare alleles that could be associated with important agricultural traits. In addition, drought tolerance index has been reported to be a reliable parameter for assessing crop tolerance to water-deficit conditions. Therefore, the objectives of this study were to evaluate the drought tolerance index for plant growth habit, plant maturity, flowering time, 100-seed weight, and grain yield in a MAGIC cowpea population, to conduct genome-wide association study (GWAS) and identify single nucleotide polymorphism (SNP) markers associated with the drought tolerance indices, to investigate the potential relationship existing between the significant loci associated with the drought tolerance indices, and to conduct genomic selection (GS). These analyses were performed using the existing phenotypic and genotypic data published for the MAGIC population which consisted of 305 F8 recombinant inbred lines (RILs) developed at University of California, Riverside. The results indicated that: (1) large variation in drought tolerance indices existed among the cowpea genotypes, (2) a total of 14, 18, 5, 5, and 35 SNPs were associated with plant growth habit change due to drought stress, and drought tolerance indices for maturity, flowering time, 100-seed weight, and grain yield, respectively, (3) the network-guided approach revealed clear interactions between the loci associated with the drought tolerance traits, and (4) the GS accuracy varied from low to moderate. These results could be applied to improve drought tolerance in cowpea through marker-assisted selection (MAS) and genomic selection (GS). To the best of our knowledge, this is the first report on marker loci associated with drought tolerance indices in cowpea.

## Introduction

Cowpea [*Vigna unguiculata* (L.) Walp.] is a diploid legume (2n = 2x = 22) grown for its relatively high amount of seed protein^1^. Cowpea cultivation exists in Asia, Oceania, the Middle East, southern Europe, Africa, southern USA, and Central and South America^2^. Cowpea has also been shown to be nutrient-dense. Cowpea seeds consisted on average of 6.8 iron, 4.1 zinc, 1.5 manganese, 510.0 phosphorus, and 1430.0 potassium, in mg per 100-g seed (Frota et al.^3^). Cowpea consumption has proven to promote health due to the high amount of antioxidants found in cowpea seeds^4,5^. In addition to grain nutritional values, cowpea biomass can be used for animal feed and cover–crop production^6^. Cowpea was grown on more than 11 million hectares worldwide, and over 70% of that came from Africa with Nigeria being the top producer^7^. Among developed countries, the United States has the greatest potential for exporting cowpea with the highest average cowpea yield per hectare^8^.

Cowpea cultivation in many parts of the world is usually rain-dependent and water shortage during cowpea developmental and growth stages could be detrimental to cowpea production^9^. Evidence of the negative effects of drought stress on cowpea has been reported in areas where cowpea is cultivated^10,11^. Even though cowpea is one of the most drought-tolerant legumes, some cultivars with desirable agronomic traits were found to be sensitive to water-deficit conditions^12^. Therefore, cowpea breeding programs aiming at improving drought tolerance is still required. Breeding for drought tolerance can make use of good understanding of the genetic control for drought tolerance. With an estimated genome size of 620 Mb^13^, cowpea could be used as an excellent model crop for drought tolerance-related studies in legume research. The relatively small genome size of cowpea would allow for a rapid and efficient identification of genes contributing to drought tolerance. Drought tolerance in cowpea is a complex trait, which involves sophisticated interactions between genes^11^, so identifying genes for drought tolerance has been a challenge. In addition, incorporating the genetic finding into breeding programs for improving drought tolerance of the existing cowpea elite culticars would be limited by narrow genetic base through biparental crosses. This could be addressed by performing drought tolerance research on a multi-parent advanced generation intercross (MAGIC) population derived from founder parents altogether having drought tolerance and other desirable agronomic traits.

Investigation the genetic architecture governing a trait of interest using a MAGIC population has recently received significant consideration. MAGIC population provides both greater diversity and a balanced allele frequency, which is critical for efficiently conducting genetic-related studies^14^. MAGIC population was first developed to dissect the genetic architecture of important traits in animals and results were promising^15^. For plants, MAGIC population has been established for *Arabidopsis thaliana*^16^, wheat^17^, rice^18^, and chickpea^19^. The genetics of yield and tolerance to abiotic stress such as drought have been successfully investigated in a MAGIC rice population^18^. Investigating the genetics of drought tolerance on a MAGIC cowpea population could be also achieved. The first MAGIC cowpea population was developed from the University of California, Riverside^20^.

This MAGIC cowpea population was phenotyped under normal and restricted irrigation conditions in California and genotyped with 51,128 SNPs using the Illumina Cowpea Consortium Array^21^. Markers associated with drought tolerance and agronomic traits such as flowering time, growth habit, and maturity were investigated based upon QTL analysis. Genetic maps, recombination frequency analysis, and significant QTLs related to the aforementioned traits were established for the MAGIC cowpea population^20^. This study was complemented using a genome-wide association study (GWAS) approach^22^. GWAS provides a greater mapping resolution over QTL mapping and efficiently permits the discovery of new genes^23,24^. However, the drought tolerance index trait, which is the relative change of the trait values due to drought stress^25,26^, was not investigated in this MAGIC cowpea population. Investigating the genetic architecture of the drought tolerance indices could lead to the discovery of new significant loci associated with drought tolerance in cowpea. In addition, the analysis can be further enhanced using genomic selection. Predictive breeding involving genomic selection has become more and more popular since it is cost-effective and provides breeders with a rapid genetic gain per unit of time^27^. Genomic selection has been reported to be highly efficient in investigating the genetic architecture of complex trait such as drought tolerance^28^. Therefore, the objectives of this study were to conduct a GWAS and GS for the drought tolerance indices, to identify SNP markers associated with drought tolerance indices, and to estimate the GS accuracy in predicting drought tolerance indices in a MAGIC cowpea population.

## Materials and methods

### MAGIC population development and genotyping

The population was developed and genotyped by Huynh et al.^20^. In brief, the population was derived from crosses between 8 genetically diverse parents (IT89KD-288, IT84S-2049, CB27, IT82E-18, SuViTa_2, IT00K-1263, IT84S-2246, and IT93K-503-1), which were cultivars and breeding lines from Burkina Faso, Nigeria, and the United States. IT93K-503-1 was an advanced drought-tolerant line developed by IITA, Nigeria^29^. The remaining parents harbored a combination of important agronomic traits such as resistance to *Striga*, fungi, bacteria, viruses, foliar thrips, root-knot nematode, and heat stress^29–35^. The first crosses were done in early 2011. The resulting MAGIC population consisted of a total of 305 F_8:10_ RIL lines. These RIL lines and founder parents were genotyped using of total of 51,128 SNPs form the Illumina Cowpea Consortium Array^21^. After SNP filtering, a total of 32,059 high-quality SNPs were retained (missing data <10%, heterozygosity <10%, and minor allele frequency >5%).

### Growing conditions and phenotyping

Phenotypic data from field phenotyping experiments of MAGIC RILs and parents were published in previous report by Huynh et al.^20^. In brief, the experiments were conducted in 2 years in two locations with two replicates (block) (1) during summer, from June (14.5 h) to September (12.8 h), at UCR-CES (33.97° N, 117.34° W) and (2) during autumn, from September (12.8 h) to December (9.9 h), at CVARS (33.52° N, 116.15° W). In 2015, the population was planted in two blocks that received different watering regimes (full irrigation and restricted irrigation) and were separated by a six-row buffer (5 m). In 2016, the two experiments (full irrigation and restricted irrigation) were repeated on adjacent field blocks. For the restricted irrigation, fields were watered to field capacity before sowing and no irrigation was done until harvest. Under the well-watered regime, water was supplied before planting and field irrigation was conducted when needed. Each set of repeated trials at UCR-CES and CVARS was considered as a randomized complete block design, with each field site per season receiving one watering treatment corresponding to a block. The plant growth habit, flowering date, maturity date, grain yield, and 100-seed weight were measured under both full and restricted irrigations. The planting areas were irrigated to field capacity before planting and restricted water regime was achieved by withholding water on the 2-week-old cowpea plants. Flowering dates were recorded when 50% of plants within a plot had flowered. Plant growth habit was rated based on a 1 to 6-scale (1: acute erect, 2: erect, 3: semi-erect, 4: indeterminate, 5: semi-prostrate, and 6: semi-prostrate). Maturity date was recorded when over 95% of pods within a row were dry. Grain yield and 100-seed weight were recorded upon harvest.

### Data analysis

In order to assess the effects of restricted irrigation on the aforementioned agronomic traits, drought stress tolerance index was computed and defined as described in Saad et al.^26^.

$${\mathrm{Tolerance}}\,{\mathrm{index}} = 100 \times \left( {Y_{{\mathrm{restricticed}}\,{\mathrm{irrigation}}}/Y_{{\mathrm{full}}\,{\mathrm{irrigation}}}} \right)$$

where Y_restricticed irrigation_ represented flowering time, maturity, grain yield, and 100-seed weight under restricted irrigation and Y_full irrigation_ referred to flowering time, maturity, grain yield, and 100-seed weight under full irrigation treatment. Changes in plant growth habit were quantified using a binary approach (1: no change in plant growth habit between full irrigation and restricted irrigation and 9: otherwise). Data were visualized using the ‘MASS’ package of R® v.3.6.1^36^.

Pearson’s correlation coefficients between the quantitatively evaluated traits were calculated using R® v.3.6.1 and the association between the qualitative trait (change in growth habit) and the quantitatively evaluated traits was investigated through a univariate logistic regression, which was run in R® v.3.6.1 as well. The logistic regression model was the following.

$${\mathrm{log}}\left[ {\pi /\left( {1 - \pi } \right)} \right] = \beta _0 + \beta _iX_i$$

where π was the probability of success of an event from the conditional binomial distribution Y|N~*Bin*(N, π) with Y being the number of genotypes having change in plant growth habit under drought stress and N being the total number of genotypes, β_0_ was the intercept, β_i_ was the effect of the ith covariate on the binomial response, X_i_ denoted the ith covariate corresponding to each trait i={1: tolerance index for plant maturity, 2: tolerance for flowering time, 3: tolerance index for 100-seed weight, and 4: tolerance index for grain yield}.

### Genome-wide association study

A Bayesian Information and Linkage Disequilibrium Iteratively Nested Keyway (BLINK) model was used to conduct GWAS. The BLINK was run using in R® v.3.6.1 using the package ‘BLINK’^37^. Previous studies have shown that BLINK allowed for efficiently discovering SNPs highly associated with traits of interest over other models^37^. SNPs with a LOD greater than 3 were declared significant^38^.

BLINK was a modified and improved version of Fixed and Random Model Circulating Probability Unification (FarmCPU). FarmCPU iteratively run both a fixed effect model (FEM) and a random effect model (REM). A major assumption when running FarmCPU was the even distribution of markers within the genome, which could be easily violated. In BLINK, this assumption was relaxed by using the information from a linkage disequilibrium (LD) analysis. The REM part of FarmCPU was replaced by a second FEM in BLINK, making the running time shorter. The two FEM models used in BLINK were the following

$${\mathrm{FEM}}\left( 1 \right):y_i = M_{i1}b_1 + M_{i2}b_2 + \ldots + M_{ik}b_k + M_{ij}d_j + e_i$$

$${\mathrm{FEM}}\left( 2 \right):y_i = M_{i1}b_1 + M_{i2}b_2 + \ldots + M_{ij}b_j + e_i$$

with y_i_ being the phenotypic data from the ith sample; M_i1_,M_i2_b_2_, …, M_ik_ the genotypes of k pseudo QTNs, which were initially empty and with effects b_1_, b_2_, …, b_k_, respectively; M_ij_ being the jth genetic marker of the ith sample; and e_i_ being the residual having a distribution with mean zero and a variance σ^2^_e_. In this study, we focused on the SNPs associated with the tolerance index trait. However, we rerun the traits investigated by Huynh et al.^20^ and Olatoye et al.^22^ using BLINK and the SNPs identified for these traits were analyzed in the network analysis section. LD heatmaps were established in R® v.3.6.1 using the package ‘LDheatmap’^39^.

### Association network

A network-guided association analysis was conducted to investigate the significant loci that were associated with two or more traits. The algorithm used for constructing the network was similar to that of established by Fang et al.^40^ with slight modifications. The nodes in the network corresponded to the traits and the significant SNPs associated with each trait. The traits investigated by Huynh et al.^20^ and Olatoye et al.^22^ were represented by solid circles, whereas the tolerance index traits were visualized by solid diamonds. The SNPs associated with each trait were denoted using solid dark grey circles. The size of each trait node was fixed, whereas the size of each SNP node was proportional to its LOD value that was obtained from GWAS. The bigger the SNP node the higher its LOD. The edge of the network was represented using solid dark lines linking the SNP and trait nodes. The attribute of the edge between a pair of SNPs was proportional to the pairwise LD *r*^*2*^ between the two SNPs, which was estimated using PLINK^41^. The attribute of the edge between a SNP node and a trait node was fixed. No edges were used between trait nodes. The network was designed using Cytoscape v. 3.7.2^42^. A network was established when a SNP was associated with two or more traits, which was easily identified using a GWAS approach. In addition, a network could be also constructed when two different SNPs were associated with two different traits, but these two SNPs were in high LD. This could not be detected with GWAS. Finally, a network was also defined when two SNPs in high LD were associated to one trait, which could be considered as epistasis^40^.

### Genomic selection

Genomic selection was carried out using all 32,059 high-quality SNPs obtained from Huynh et al.^20^. Genomic estimated breeding values (GEBVs) were estimated using a ridge regression best linear unbiased predictor model (rrBLUP)^43^. The rrBLUP model was y = WGβ + ε where y was the vector phenotype, β indicated the marker effect with β~*N*(0, Iσ^2^_β_), W corresponded to the incidence matrix relating the genotype to the phenotype, G denoted the genetic matrix, and ε was the random error. The solution for the model was β^=(Z^T^Z + Iλ)^−1^Z^T^y with Z = WG. The ridge parameter used in this study was λ = σ^2^_e_/σ^2^_β_. The parameter σ^2^_e_ denoted the residual variance and $\sigma _{_\beta }^2$ the marker effect variance. rrBLUP was conducted in R® v.3.6.1 using the package ‘rrBLUP’^44^.

Genomic estimated breeding values (GEBVs) were estimated using a training population randomly chosen from the MAGIC population^45^. Since the genotypes with missing data could impact the results, they were removed prior to conducting genomic selection, leaving with a total of 249 cowpea genotypes for the analysis. Genomic selection was conducted using a two-, three-, four-, five-, six-, seven-, and eight-fold cross validation corresponding to a training/testing set of 125/124, 166/83, 186/63, 199/50, 207/42, 213/36, and 217/32, respectively. The training and testing sets were two disjoint groups. The training population was used to fit the model and the testing population was used to assess the accuracy of the model. A total of 100 replications were used for each cross-validation level. Genomic selection accuracy corresponded to the Pearson’s correlation coefficient between the GEBVs and the observed phenotypic values in the testing set^45^.

## Results

### Variation in drought tolerance

**Fig. 1:**
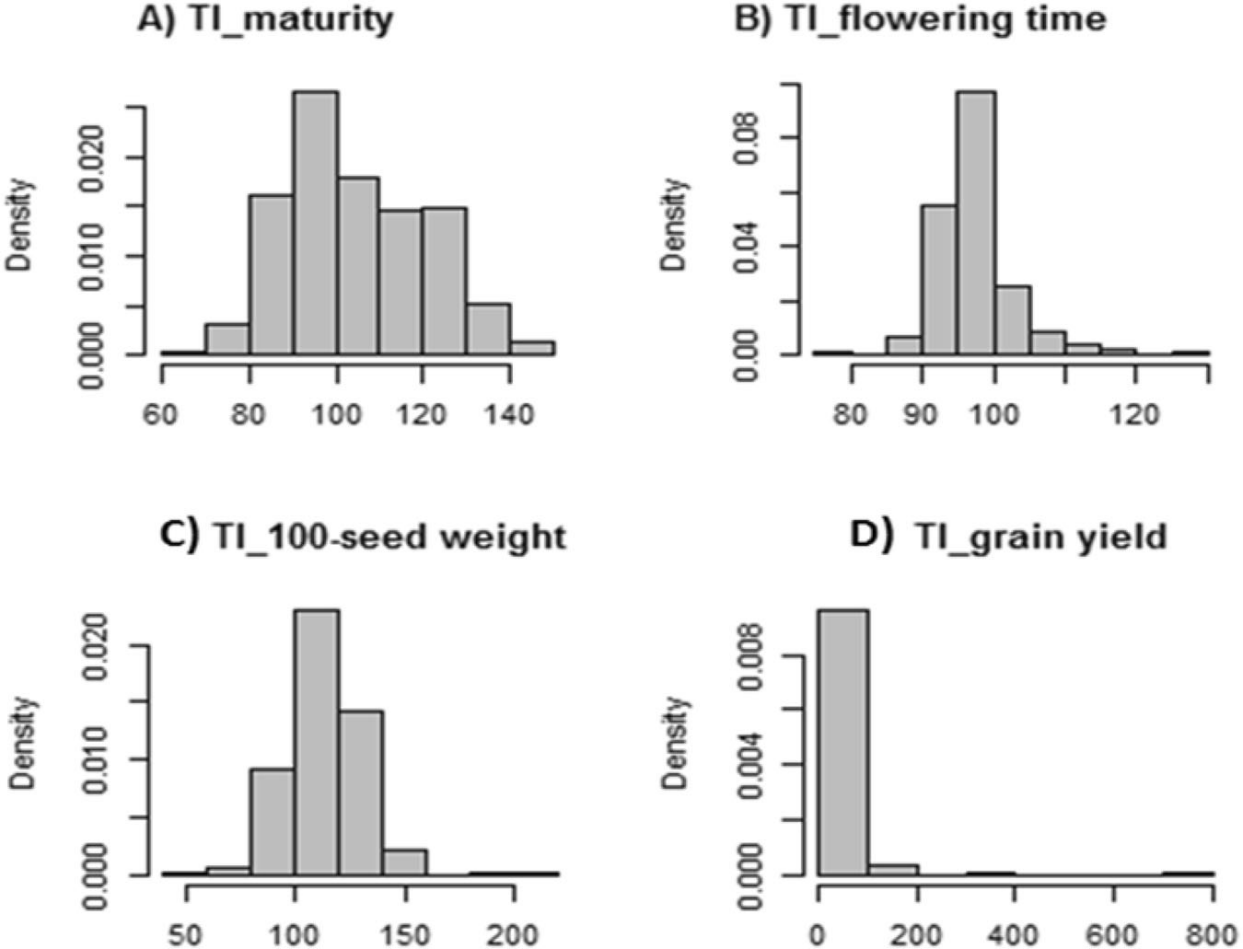
Phenotypic trait value distribution. Distribution of drought tolerance index for **A** maturity, **B** flowering time, **C** 100-seed weight, and **D** grain yield

The tolerance indexing was used to quantify the relative change in maturity due to drought stress. A tolerance index greater than 100 for plant maturity indicated that restricted irrigation made plant maturity longer, whereas a tolerance index lower than 100 suggested plant maturity being shorter due to water deficit. A large variation in tolerance index for maturity was identified among the RILs. Tolerance index was nearly normally distributed (Fig. 1a). Tolerance index ranged between 69.19 and 142.01, with an average of 104.74 and a standard deviation of 15.60 (Fig. S1).

Tolerance index for flowering time varied from 78.41 to 126.67, with an average of 97.48 and a standard deviation of 5.35. Tolerance index for flowering time was also approximately normally distributed (Fig. 1b). Tolerance index for 100-seed weight was approximately normally distributed (Fig. 1c) and ranged between 59.56 and 210.11, with an average of 113.09 and a standard deviation of 17.54 (Fig. S1).

Unlike the aforementioned parameters investigated in this study, tolerance index for grain yield was right-skewed as shown in Fig. 1d. Tolerance index ranged between 4.95 and 754.39, with an average of 41.89 and a standard deviation of 53.34, indicating that yield was negatively impacted by restricted irrigation (Fig. S1).

Plant growth habit under both full and restricted irrigations were recorded. A total of 154 RILs had a change in plant growth habit due to drought stress. Overall, the change pattern was semi-erect and indeterminate towards acute erect and erect.

Pearson’s correlation coefficients between the different tolerance indices were calculated. Overall, correlation coefficients between traits were low. A moderate and positive Pearson’s correlation coefficient was found between tolerance index for grain yield and tolerance index for 100-seed weight (*r* = 0.33). A low Pearson’s correlation coefficient was found between tolerance index for maturity and tolerance index for flowering time (*r* = 0.17). The lowest Pearson’s correlation coefficient was found between tolerance index for flowering time and tolerance index for 100-seed weight (*r* = 0.01).

A univariate logistic regression model was used to assess the relationship between change in growth habit due to drought stress and the previously assessed tolerance indices. The univariate logistic regression model was used to fit the change in growth habit to each tolerance index trait, where the growth habit was a binomial response and each tolerance index was a continuous predictor variable. The univariate model showed that all tolerance indices except for tolerance index for grain yield were insignificant. The estimate of the effects of tolerance index for plant maturity, tolerance index for grain yield, tolerance index for 100-seed weight, and tolerance index for flowering time on the change of growth habit due to drought stress were −0.009 (Z-value = −1.170, *p*-value = 0.142), 0.013 (Z-value = 2.207, *p*-value = 0.03), 0.006 (Z-value = 0.851, *p*-value = 0.395), and −0.019 (Z-value = −0.775, *p*-value = 0.438), respectively. These results indicate that there is a significant association between tolerance index for grain yield and change in growth habit to drought stress.

### Genome-wide association study

**Fig. 2:**
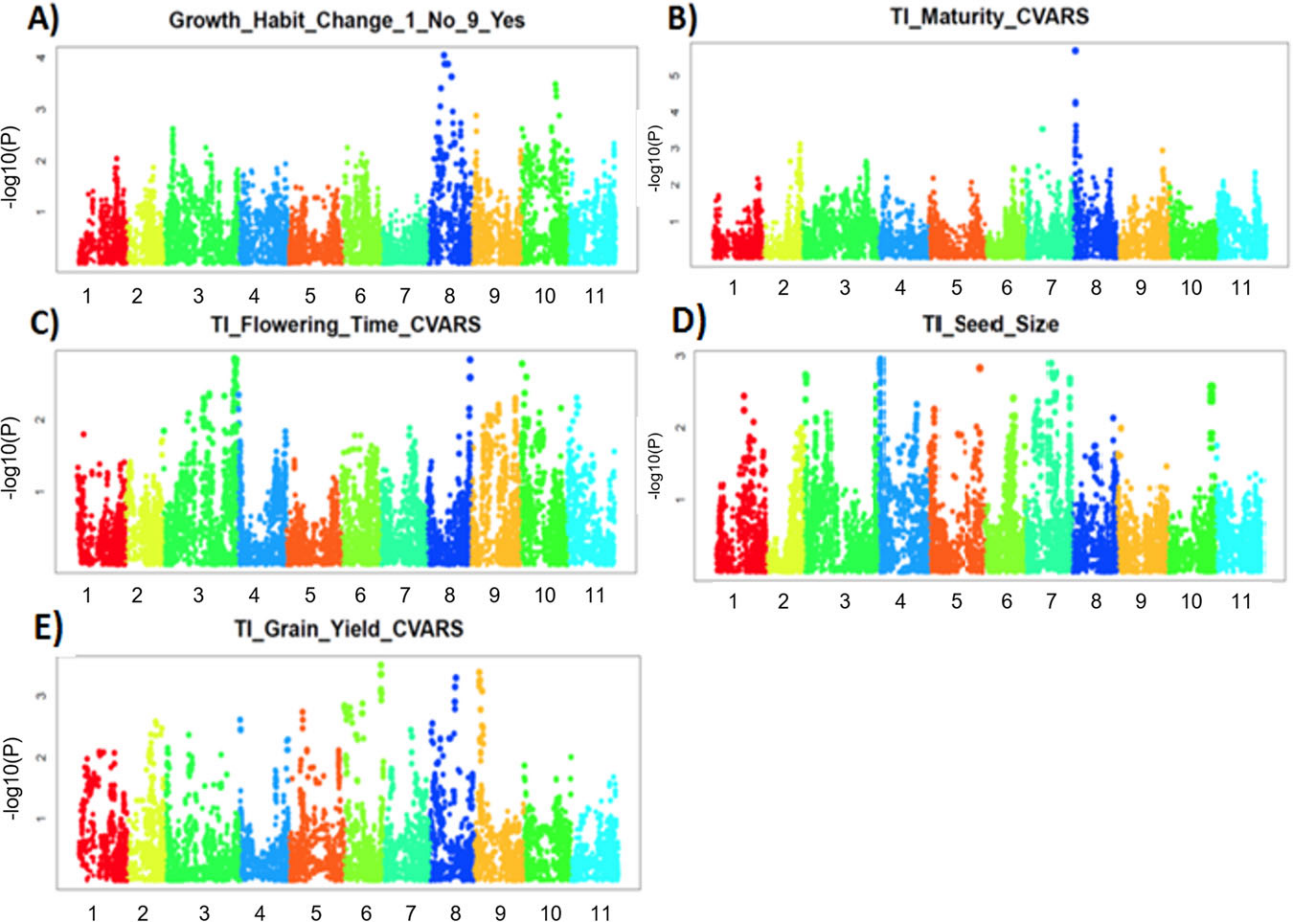
Manhattan plots showing the LOD (−log_10_(*p*-value)) for each SNP used to conduct GWAS. The *y*-axis each of Manhattan plot represents the LOD (−log_10_(*p*-value)) and the *x*-axis displays the chromosome number. Color coding on each Manhattan plot was chromosome-wise. **A** Manhattan plot for change in growth habit, **B** Manhattan plot for tolerance index for maturity, **C** Manhattan plot for tolerance index for flowering time, **D** Manhattan plot for tolerance index for seed size, and **E** Manhattan plot for tolerance index for grain yield

**Fig. 3:**
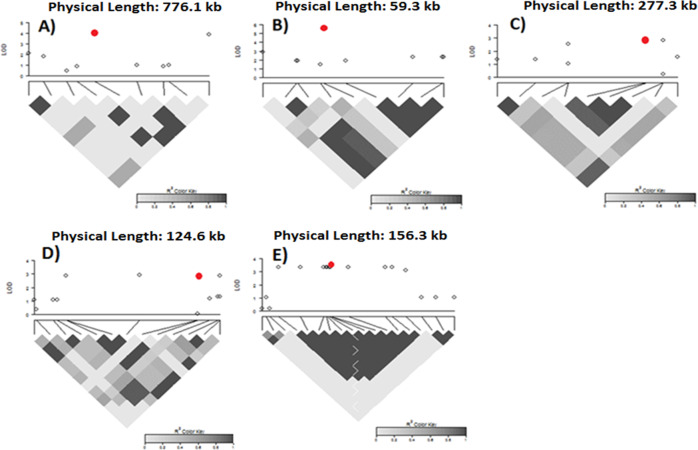
Local Manhattan plots and linkage disequilibrium (LD) heatmaps around the most significant SNP for each trait, which is shown by the red dots. For each graph, the *y*-axis of the local Manhattan represents the LOD (−log_10_(*p*-value)) of the corresponding SNP. The *x*-axis of the local Manhattan shows the physical distance (kb) between two adjacent SNPs. Below each local Manhattan plot is displayed the LD heatmap. Color coding within the LD heatmap ranges from white to black and the parameter for estimating pairwise LD was R square. The white color within the LD heatmap corresponds to an R-square value of 0, whereas the black color corresponds to an R-square value of 1. **A** Local Manhattan plot and LD heatmap on a 776.1-kb region of chromosome 8 harboring the SNP 2_26924 associated with change in growth habit, **B** Local Manhattan plot and LD heatmap on a 59.3-kb region of chromosome harboring the SNP 2_21981 associated with tolerance index for maturity, **C** Local Manhattan plot and LD heatmap on a 227.3-kb region of chromosome 3 harboring the SNP 2_06470 associated with tolerance index for flowering time, **D** Local Manhattan plot and LD heatmap on a 124.6-kb region of chromosome 4 harboring the SNP 2_11122 associated with tolerance index for seed weight, and **E** Local Manhattan plot and LD heatmap on a 156.3-kb region of chromosome 6 harboring the SNP 2_25334 associated with tolerance index for yield

GWAS was conducted to identify SNP markers associated with growth habit change, tolerance indices for maturity, flowering time, 100-seed weight, and grain yield. A total of 14 SNP markers were found to be associated with tolerance index to plant growth habit change (Table 1) (Fig. 2a). Of which, 8 were mapped on a 10.1-Mb region of chromosome 8, indicating a strong likelihood of significant loci associated with plant growth habit change under drought stress in this genomic region. The top 5 SNPs associated with plant growth habit change under drought stress were 2_26924 (LOD = 4.06, MAF = 17.67%), 2_01300 (LOD = 3.88, MAF = 17.27%), 2_10658 (LOD = 3.88, MAF = 17.27%), 2_54501 (LOD = 3.88, MAF = 17.27%), and 2_45332 (LOD = 3.88, MAF = 17.27%) (Table 1), which were all located on chromosome 8. The LD analysis around the most significant SNP showed low pairwise LD values between SNPs (Fig. 3a).

**Table 1:** Significant SNPs associated with growth habit change, tolerance indices for plant maturity, flowering time, and 100-seed weight with their respective LOD (−log_10_(*p*-value)) value, and MAF (minor allele frequency)

Traits	SNP	Chromosome	Position (bp)	LOD	MAF(%)
Growth habit change	2_40797	8	10549370	3.06	12.05
	2_42112	8	10601329	3.06	12.05
	2_42607	8	11012105	3.41	31.33
	2_26924	8	13771284	4.06	17.67
	2_01300	8	14264077	3.88	17.27
	2_10658	8	15346859	3.88	17.27
	2_54501	8	16564006	3.88	17.27
	2_45332	8	16871228	3.88	17.27
	2_06275	8	17354751	3.88	17.27
	2_43529	8	20159451	3.64	17.67
	2_40435	8	20618849	3.64	17.67
	2_50806	10	29754489	3.49	12.20
	2_26782	10	30148065	3.38	13.25
	2_38918	10	30517553	3.25	13.31
Tolerance index for maturity	2_16403	2	32138108	3.13	42.17
	2_45148	2	32146045	3.13	42.17
	2_55009	7	14098180	3.54	13.65
	2_51274	7	14976910	3.54	13.65
	2_21981	8	1801037	5.68	20.08
	2_10862	8	1929122	3.20	33.33
	2_10861	8	1929370	3.20	33.33
	1_0806	8	1950113	3.00	32.93
	2_21676	8	1965506	3.00	32.93
	2_21804	8	1970485	3.00	32.93
	2_23871	8	1980059	3.00	32.93
	2_23870	8	1980643	3.00	32.93
	2_44136	8	1985249	3.00	32.93
	2_14976	8	2006627	4.23	28.92
	2_40337	8	2013873	4.27	28.34
	2_14158	8	2338417	3.63	33.33
	2_16735	8	2361920	3.47	32.93
	2_41533	8	2384266	3.34	33.20
Tolerance index for flowering time^a^	2_06470	3	62407410	2.84	12.45
	2_52919	3	62409665	2.84	12.45
	2_06137	3	62434051	2.84	12.45
	1_0946	3	63722355	2.83	11.65
	2_27706	8	37928961	2.83	19.68
Tolerance index for 100-seed weight^a^	2_11122	4	1483784	2.95	11.34
	2_03731	4	1523145	2.89	10.84
	2_14932	4	1548833	2.89	10.84
	2_34365	4	1549730	2.89	10.84
	2_07882	4	1556026	2.89	10.84

^a^No SNPs having an LOD value greater than the chosen threshold (3) were found so that the top 5 SNPs with the highest LOD value are presented

The results indicated a total of 18 SNPs associated with tolerance index for maturity (Table 1) (Fig. 2b). Of which, 14 were found on a 584-Kb region of chromosome 8. A small portion of this region overlapped with the 10.1-Mb region found for plant growth habit change under drought stress. The remaining SNPs were located on chromosomes 2 and 7. The top 5 SNPs with the highest LOD value were 2_21981 (LOD = 5.68, MAF = 20.08%), 2_40337 (LOD = 4.27, MAF = 28.34%), 2_14976 (LOD = 4.23, MAF = 28.92%), 2_14158 (LOD = 3.63, MAF = 33.33%), and 2_51274 (LOD = 3.54, MAF = 13.65%) (Table 1). The region in the vicinity of the SNP with the highest LOD value indicated a moderate LD (Fig. 3b). In addition, no SNPs located within the 30-kb region flanking the most significant SNP, 2_21981, had an LOD greater than the declared threshold (3) (Fig. 3b).

The discrepancy in change in flowering time between full irrigation and restricted irrigation was also assessed using tolerance index for flowering time. However, no SNPs exceeding the LOD threshold (3) were found. We only reported the top 5 SNPs, 2_06470 (LOD = 2.84, MAF = 12.45%), 2_52919 (LOD = 2.84, MAF = 12.45%), 2_06137 (LOD = 2.84, MAF = 12.45%), 2_27706 (LOD = 2.83, MAF = 19.68%), and 1_0946 (LOD = 2.83, MAF = 11.65%) that the GWAS analysis suggested for tolerance index for flowering time (Table 1) (Fig. 2c). One of these SNPs were located on chromosome 8 (Fig. 2c). However, this SNP was not located within the significantly associated loci identified for plant growth habit change and tolerance index for plant maturity. The region harboring the most significant SNP, 2_06470, had a high LD (Fig. 3c).

The results did not show any SNPs having an LOD greater than the threshold (3) for tolerance index for 100-seed weight under restricted irrigation. We just reported the top 5 SNPs having the highest LOD values (Table 1). These SNPs were 2_11122 (LOD = 2.95, MAF = 11.34%), 2_03731 (LOD = 2.89, MAF = 10.84%), 2_14932 (LOD = 2.89, MAF = 10.84%), 2_34365 (LOD = 2.89, MAF = 10.84%), and 2_07882 (LOD = 2.89, MAF = 10.84%). These SNPs were all found on chromosome 4 (Fig. 2d). Among all traits evaluated in this study, tolerance index for grain yield had the highest number of significant SNPs. Our data suggested indicated a total of 35 SNPs associated with tolerance index for grain yield (Table 2) (Fig. 2e). Of which, 26 were mapped on a 566.5-Kb region of chromosome 6, 7 on a 2.5-Mb region of chromosome 8, and 2 on a 703-Kb region of chromosome 8 (Table 2). These regions could harbor significant loci associated with tolerance index for grain yield under drought stress in cowpea. The top 5 SNPs with the highest LOD value were 2_25334 (LOD = 3.51, MAF = 8.23%), 2_51818 (LOD = 3.38, MAF = 12.85%), 2_31565 (LOD = 3.35, MAF = 9.64%), 2_19053 (LOD = 3.35, MAF = 9.64%), and 2_33474 (LOD = 3.35, MAF = 9.64%). The LD heatmap shown in Fig. 3e revealed an independent LD block, which contained the most significant SNP associated tolerance index for grain under drought stress. This LD pattern was not identified for traits such as change in plant growth habit, tolerance index for maturity, flowering time, and 100-seed weight. In addition, there is a lack of overlaps between the significant SNPs across different traits, indicating that drought stress is a complex mechanism.

**Table 2:** Significant SNPs associated with tolerance index for grain yield with their respective LOD (−log_10_(p-value)) value, and MAF (minor allele frequency)

Traits	SNP	Chromosome	Position (bp)	LOD	MAF(%)
Tolerance index for grain yield	2_31564	6	32057972	3.35	9.64
	2_31565	6	32058239	3.35	9.64
	2_30808	6	32061499	3.35	9.64
	2_19053	6	32061827	3.35	9.64
	2_33474	6	32070478	3.35	9.64
	2_28131	6	32077832	3.35	9.64
	2_28570	6	32088910	3.09	9.80
	2_10632	6	32089786	3.35	9.64
	2_13247	6	32107028	3.35	9.64
	2_18126	6	32147410	3.35	9.64
	2_14728	6	32165112	3.35	9.64
	2_02004	6	32184138	3.35	9.64
	2_25332	6	32186496	3.35	9.64
	2_33745	6	32186893	3.35	9.64
	2_25331	6	32188321	3.35	9.64
	2_25334	6	32189396	3.51	8.23
	2_25333	6	32189710	3.35	9.64
	2_30533	6	32204324	3.35	9.64
	2_31969	6	32234310	3.35	9.64
	2_32622	6	32239677	3.35	9.64
	2_50666	6	32250975	3.11	9.92
	2_21574	6	32454860	3.05	9.64
	2_29076	6	32461137	3.05	9.64
	1_0823	6	32612013	3.05	9.64
	2_15103	6	32612013	3.05	9.64
	2_21155	6	32624482	3.05	9.64
	2_53988	8	21904122	3.15	11.24
	2_46582	8	22607265	3.29	11.65
	2_01303	9	4760699	3.24	13.25
	2_51818	9	4789752	3.38	12.85
	2_35898	9	4877591	3.16	13.65
	2_23949	9	5346101	3.26	14.46
	2_23950	9	5347304	3.26	14.46
	2_11952	9	5364438	3.26	14.46
	2_34102	9	7298753	3.08	9.79

### Network-guided GWAS

An association network was established in order to investigate the possible interactions between loci, which were found to be significantly associated with each tolerance index trait measured in the MAGIC cowpea population under drought stress. In addition, trait-associated loci reported in Huynh et al. (2018) and Olatoye et al. (2019) were also incorporated into the network. The network was designed to be an extension of the GWAS analysis in such a way that the SNPs in high LD (Linkage disequilibrium) with the SNP having the highest LOD value for each trait were used to perform the analysis.

**Fig. 4:**
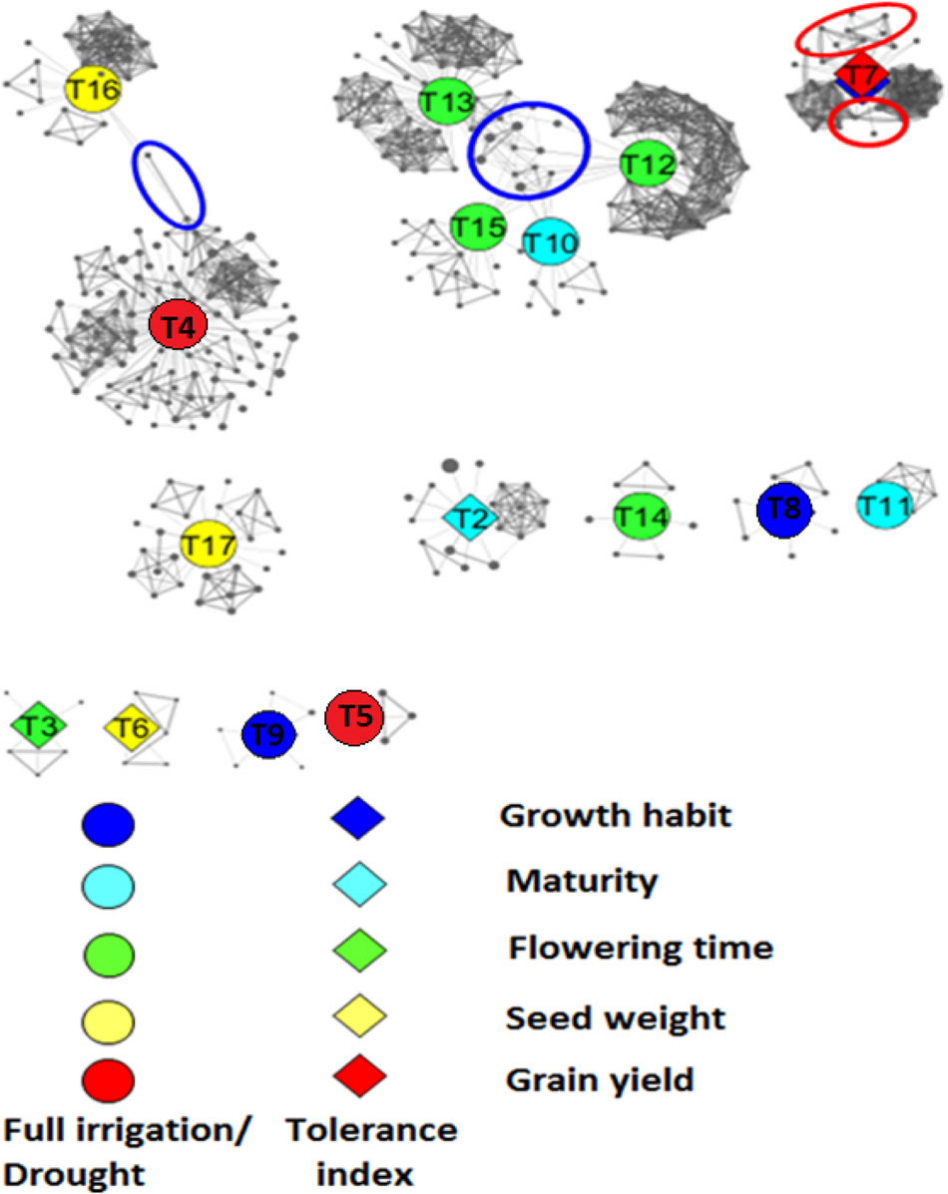
Association networks displaying the tolerance indices of growth habit, maturity, flowering time, seed weight, and grain yield under drought stress in a MAGIC cowpea population. The solid circles represent the traits evaluated under full irrigation and drought stress conditions. The solid diamonds correspond to the tolerance indices for different traits under drought stress. The solid dark gray circles show the significant SNPs associated with each trait. The size of each SNP node is proportional to its LOD value. Edges between nodes are represented by solid black lines. Edges with similar size are used to link each trait node to each SNP node. Edges with different size are used to link different SNP nodes. The link power of the edge between each SNP node was the R-square linkage disequilibrium (LD) value between the two SNPs. The empty red circles represent the significant loci associated with the tolerance index trait values. The empty blue circles display the epistatic loci reported by Olatoye et al. (2019). The legend corresponding to each trait node was the following: T1 = Tolerance index for growth habit change, T2 = tolerance index for plant maturity, T3 = tolerance index for flowering time, T4 = grain yield under full irrigation, T5 = grain yield under drought stress, T6 = tolerance index for 100-seed weight, T7 = tolerance index for grain yield, T8 = growth habit under full irrigation, T9 = growth habit under drought stress, T10 = maturity under full irrigation, T11 = maturity under drought stress, T12 = flowering time under full irrigation, T13 = flowering time under drought stress, T14 = flowering time under full irrigation at UCR, T15 = flowering time under drought stress at UCR, T16 = seed weight under full irrigation, and T17 = seed weight under drought stress. Tolerance index for flowering time at UCR was not calculated since the experiments were conducted under two different seasons at this location

The network-guided GWAS indicated 12 independent subnetworks as shown in Fig. 4. The solid diamonds on Fig. 4 showed the tolerance index trait, whereas the solid circles indicated to traits investigated by Huynh et al.^20^ and Olatoye et al.^22^. The solid dark gray circles surrounding each trait corresponded to the SNPs. These results provided a clear visualization of the genetic architecture affecting each trait and suggested that some traits were likely to be correlated at the genetic level, whereas other traits were more genetically independent from the others. Traits such as tolerance index for plant maturity (T2), tolerance index for flowering time (T3), and tolerance index for 100-seed weight (T6) had independent significant loci (Fig. 4), suggesting that these traits could have independent drought tolerance mechanism and should be investigated separately when studying drought tolerance in cowpea.

The network-guided GWAS revealed interacting loci for change in growth habit and tolerance index for grain as shown by the solid blue and red diamonds, respectively, in the upper right-corner of Fig. 4. The two interacting loci were highlighted using the empty red circles. This result suggested that tolerance index for grain yield and change in growth habit had common significantly associated loci. Interestingly, this network existing between loci affecting tolerance index for grain yield and change in growth habit was not identified via GWAS alone, indicating that a network analysis could complement GWAS to provide additional information to investigate the genetics of drought tolerance in cowpea.

The network analysis revealed common loci between traits, which were identified using GWAS. These findings showed that GWAS and network analysis could be used to validate each other. In addition, the network analysis displayed epistatic loci for each trait evaluated in this study. Significant epistatic loci, shown by the interactions between SNPs within each trait, were found for tolerance index for grain yield, change in growth habit, and tolerance index for plant maturity (Fig. 4).

### Genomic selection

**Fig. 5:**
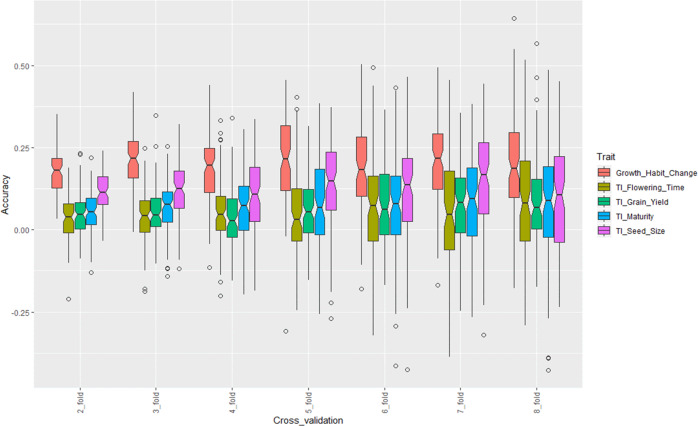
Genomic selection accuracy using a ridge regression best linear unbiased predictor model (rrBLUP) for change in plant growth habit, tolerance index for flowering time, grain yield, plant maturity, and 100-seed weight. Genomic selection was conducted using a 2-fold, 3-fold, 4-fold, 5-fold, 6-fold, 7-fold, and 8-fold cross validation. The *y*-axis of the figure represents the accuracy of genomic selection at each cross-validation fold for each trait

Genomic selection was conducted using a ridge regression best linear unbiased predictor model (rrBLUP) for change in plant growth habit due to a restricted irrigation, tolerance index for plant maturity, tolerance index for flowering time, tolerance index for 100-seed weight, and tolerance index for grain yield. The accuracy of genomic selection was evaluated under different cross-validation folds. Overall, genomic selection was low for almost all traits. At each cross-validation fold, variation in genomic selection accuracy was identified between each tolerance index trait (Fig. 5). Genomic selection accuracy for change in growth habit was highest regardless of the training population size. The average genomic selection accuracy for change in growth habit was 0.18, 0.21, 0.19, 0.21, 0.19, 0.21, and 0.19 at 2-fold, 3-fold, 4-fold, 5-fold, 6-fold, 7-fold, and 8-fold cross validation, respectively. Genomic selection accuracy for tolerance index for 100-seed weight was second highest at 2-fold (0.12), 3-fold (0.12), 5-fold (0.13), 6-fold (0.12), and 7-fold (0.15) cross validation (Fig. 5). The increase in training population size seemed to be more favorable to improving the genomic selection accuracy of tolerance for 100-seed weight than enhancing the genomic selection accuracy for tolerance index for grain yield. The lowest genomic selection accuracy was recorded for tolerance index for flowering time (2-fold: 0.05, 3-fold: 0.07, 4-fold: 0.07, 5-fold: 0.08, 6-fold: 0.08, 7-fold: 0.08, and 8-fold: 0.08) and for tolerance index for grain yield (2-fold: 0.05, 3-fold: 0.05, 4-fold: 0.05, 6-fold: 0.08, 7-fold: 0.08, and 8-fold: 0.08) (Fig. 5).

## Discussion

Change in plant growth habit, tolerance index for plant maturity, tolerance index for flowering time, tolerance index for 100-seed weight, and tolerance index for grain yield were evaluated to quantify the relative tolerance to drought stress of the MAGIC cowpea population used for this study. Tolerance index has been used for efficiently assessing plant stress tolerance in previous studies^25,26^. Our results indicated large variation in tolerance index trait among the cowpea genotypes evaluated in this study, suggesting that this population is genetically diverse and could be used to enhance drought tolerance in a cowpea breeding program. However, the Pearson’s correlation coefficients analysis between the tolerance index traits were low, indicating that drought tolerance mechanism between the tolerance index traits could be independent. These results were in-line with previously reported studies on the possible independent mechanisms affecting drought tolerance in cowpea^12,46^. The logistic regression model of change in plant growth habit on tolerance index for grain yield was significant, which suggested an association between these two traits. This funding was critical since it established a link between growth habit and tolerance to grain yield reduction due to drought stress in cowpea. Additional studies will be required to investigate the pathways that could lead to the association between plant growth habit and tolerance to the decrease in grain yield under restricted irrigation in cowpea.

Genome-wide association study (GWAS) was conducted to identify SNP markers associated with the tolerance index traits. The number of significant SNPs varied between the tolerance index traits. As expected, tolerance index for grain yield had the highest number of SNP markers, indicating that a large number of loci could contribute to maintaining high yield in cowpea genotypes subjected to restricted water supplies. These results were in agreement with previous investigations reporting grain yield being a polygenic trait^47,48^. The MAGIC cowpea population used in this study was first investigated by Huynh et al.^20^ and Olatoye et al.^22^. They conducted GWAS for flowering time, plant maturity, plant growth habit, 100-seed weight, and grain yield under full irrigation and restricted irrigation, respectively. In this study, we improve their analysis by assessing the drought tolerance of each individual within the cowpea MAGIC population using the tolerance index formula^25,26^. The GWAS was reanalyzed based on tolerance indices. Results indicated the discovery of new loci affecting the tolerance index traits. These loci were not identified by Huynh et al.^20^ and Olatoye et al.^22^. Therefore, our findings complement the approach conducted by Huynh et al.^20^ and Olatoye et al.^22^ to investigate drought tolerance in the MAGIC cowpea population. In addition, we integrated the reported loci identified by Huynh et al.^20^ and Olatoye et al.^22^ into a network that displayed the newly discovered loci for tolerance index. The network analysis suggested a clear independency between the different loci, which supported our previous claim on the independency of drought tolerance mechanism affecting different traits in cowpea. Olatoye et al.^22^ investigated the epistatic interactions between loci affecting the traits evaluated by Huynh et al.^20^. These interactions were found using a network-guided approach as shown in Fig. 4, which suggests that the algorithm we used to establish the network analysis was valid. One of the significant findings from this current study was the discovery of two loci affecting both change in plant growth habit and tolerance index for grain yield (Fig. 4). These loci were rich in transmembrane amino acid transporters and MYB-transcription factors. The role of biomolecule transporters in regulating plant response to water-deficit conditions has been well-documented. Jarzyniak and Jasiński^49^ stated that the transmembrane transporters significantly affect stomatal and cuticular activities during drought stress in plant. These biomolecules could also affect root responses under water-deficit conditions. MYB-transcription factors have been shown to assist plant with withstanding drought stress. The expression of MYB-transcription factors have been correlated with the capability of plants to survive under drought conditions^50–52^. These findings showed that the approach we used for investigating the genetic architecture of drought tolerance in this MAGIC cowpea population could efficiently target candidate genes that are relevant to drought tolerance.

Genomic selection for change in growth habit, drought tolerance index for flowering time, plant maturity, 100-seed weight, and grain yield was conducted using a ridge regression best linear unbiased predictor model. Genomic selection has been proven to be effective when dealing with complex traits such as drought tolerance^28,53^. In this study, genomic selection accuracy varied from low to moderate. This could be attributed to the complexity of the drought tolerance traits. Olatoye et al.^22^ evaluated the prediction accuracy of flowering time, maturity date, and seed size under full irrigation and restricted irrigation, respectively, from the data generated by Huynh et al.^20^ and using the same MAGIC population reported in this current work. The prediction accuracy was higher for flowering time, maturity date, and seed size under full irrigation and restricted irrigation, respectively. This could be explained by the fact that these traits were more heritable than their respective drought tolerance indices, which were calculated based on the ratio of the trait values from restricted irrigation and full irrigation, respectively. Even though the genomic selection accuracy varied from low to moderate, it can still supplement the phenotypic selection and would increase the genetic gain by at least 10%^54^.

## Conclusion

In this study, large variation in drought tolerance indices for plant growth habit, flowering time, plant maturity, 100-seed weight, and grain yield was found within the MAGIC cowpea population. New loci associated with these drought tolerance traits were identified and a network-guided strategy assisted with the discovery of overlapping significant loci associated with the drought tolerance indices. In addition, genomic selection accuracy varied from low to moderate. The results from this investigation will contribute to a better understanding of the genetic architecture governing drought tolerance in cowpea and could be used in cowpea breeding programs through marker-assisted selection and genomic selection.

## Supplementary information

TableS1

TableS2

FigS1
