# Supplementary figures and images for: Loci discovery, network-guided approach, and genomic prediction for drought tolerance index in a multi-parent advanced generation intercross (MAGIC) cowpea population
Source: Hortic Res. 2021 Feb 1;8:24. doi: 10.1038/s41438-021-00462-w (PMC7848001; doi:10.1038/s41438-021-00462-w)

## Slide 1
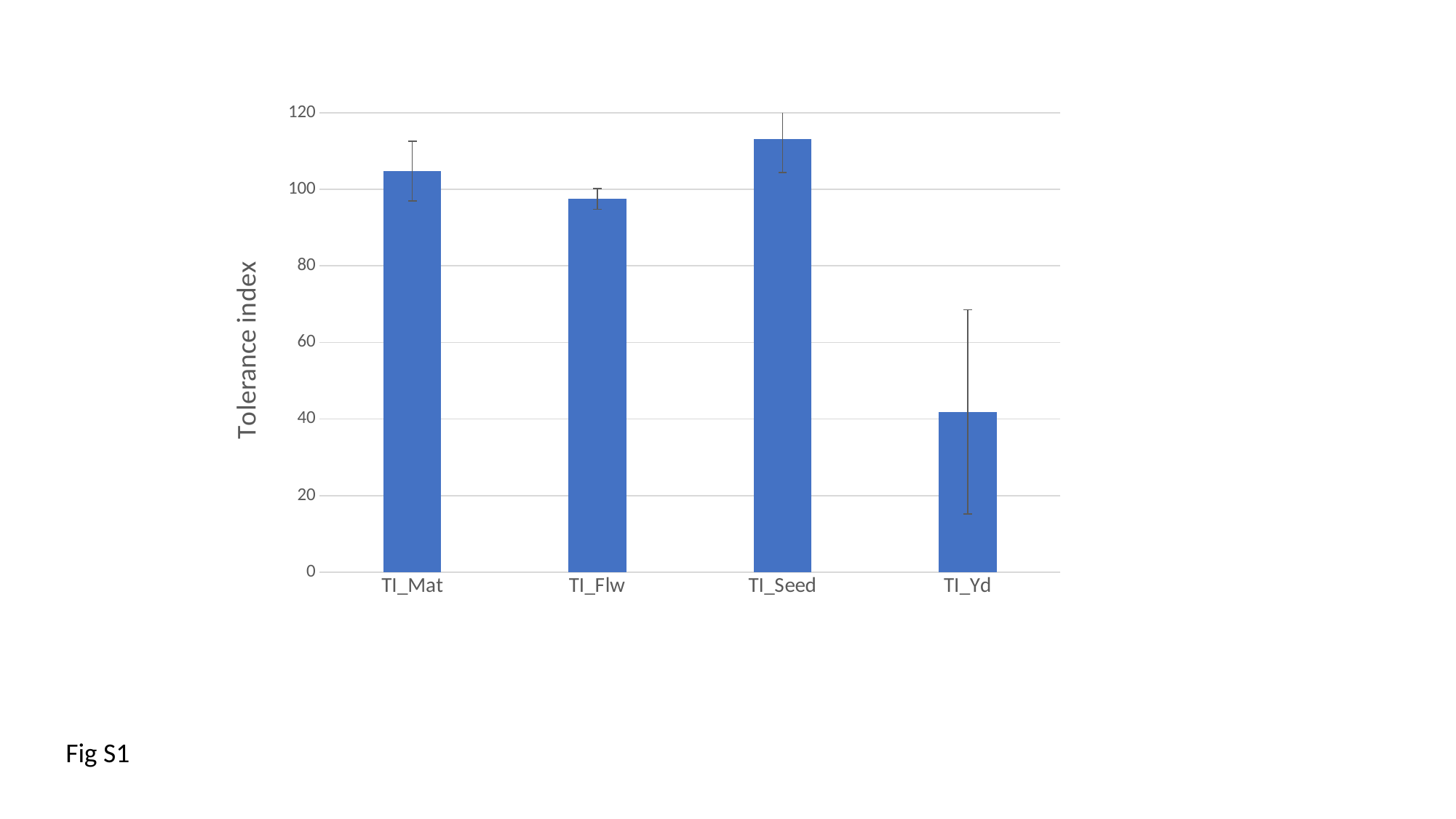

### Chart
| Category | average |
|---|---|
| TI_Mat | 104.74261044176706 |
| TI_Flw | 97.47538152610439 |
| TI_Seed | 113.09204819277106 |
| TI_Yd | 41.8915261044177 |Fig S1

Supplement: Supplementary file 3 — FigS1 [file 41438_2021_462_MOESM3_ESM.pptx]
